# Targeted Therapy in Hepatocellular Carcinoma

**DOI:** 10.4061/2011/348297

**Published:** 2011-07-12

**Authors:** Clarinda W. L. Chua, Su Pin Choo

**Affiliations:** Department of Medical Oncology, National Cancer Centre Singapore, 11 Hospital Drive, 169610, Singapore

## Abstract

Hepatocellular carcinoma (HCC) is one of the commonest cancers worldwide, as well as a common cause of cancer-related death. HCC frequently occurs in the setting of a diseased cirrhotic liver and many patients present at an advanced stage of disease. Together with a poor functional status, this often precludes the use of systemic therapy, especially conventional cytotoxic drugs. Moreover, HCC is known to be a relatively chemo-refractory tumor. There have been many targeted drugs that have shown potential in the treatment of HCC. Many clinical trials have been carried out with many more in progress. They include trials evaluating a single targeted therapy alone, two or more targeted therapy in tandem or a combination of targeted therapy and conventional chemotherapy. In this article, we seek to review some of the more important trials examining the use of targeted therapy in HCC and to look into what the future holds in terms of targeted treatment of HCC.

## 1. Introduction

Liver cancer is the sixth most common cancer worldwide, accounting for 5.7% of new cancer cases, and the third most common cause of cancer-related death [1]. The majority of cases and deaths occur in developing countries. Of the primary liver tumors in adults, hepatocellular carcinoma (HCC) is the commonest [2].

HCC frequently occurs in the setting of a diseased cirrhotic liver. It has well-defined risk factors, the most common being infections with hepatitis B virus (HBV) and hepatitis C virus (HCV). Chronic excessive alcohol consumption, environmental toxins, for example, aflatoxin B and nonalcoholic steatohepatitis (NASH), make up the rest of the main causes. The etiological factors vary by geographical locations [3]. In Africa and East Asian countries including Taiwan, China, and Korea, HBV is the main cause whereas in the West and in Japan, HCV is the main risk factor, together with other causes of cirrhosis including alcohol [3, 4].

The asymptomatic nature of a HBV and HCV carrier state, the insidious presentation of early HCC, and screening programs that are not properly defined or adhered to results in the majority of patients with HCC presenting at an intermediate or advanced state. Potentially curative strategies such as resection and transplantation as well as loco-regional therapies such as radiofrequency ablation and transarterial chemoembolization are often not possible at these stages. 

Systemic treatment with chemotherapy is not routinely employed in the treatment of advanced HCC for a variety of reasons. As HCC usually occurs in the context of a diseased cirrhotic liver, poor hepatic reserves often preclude or limit systemic chemotherapy. Also, HCC is known to be a relatively chemorefractory tumor, in part due to overexpression of drug-resistant genes including *MDR1* [5]. Trials involving chemotherapeutic agents were carried out in diverse populations, limiting their application across the board to the entire cohort of HCC patients. 

Several studies of chemotherapeutic agents have shown them to have limited activity in HCC [6–8]. Various clinical trials investigating the role of single-agent chemotherapy on the other hand have previously reported response rates from 0% to 20%. Anthracyclines, for example, doxorubicin have shown a response rate of up to 20% [9–12]; their usage, though, has been limited by elevated toxicity.

A randomized phase III study by Yeo et al. [13] reported a response rate of 21% using PIAF (cisplatin, doxorubicin, interferon, and fluorouracil) in 91 of 94 assessable patients with unresectable HCC with a median overall survival (OS) of 8.7 months. Lombardi and colleagues demonstrated a response rate of 24% with pegylated liposomal doxorubicin and gemcitabine in patients with advanced HCC [14]. In this study, one patient went on to undergo liver transplantation and another underwent surgical resection. About half of the patients were Child-Pugh B. 

Although chemotherapy in advanced HCC has been shown in various trials to have relatively significant response rates, its usage is limited by toxicities, especially in patients with poor hepatic reserves. Moreover, the phase III trial using PIAF did not show survival benefit over single agent doxorubicin alone. 

The poor prognosis of patients with advanced or metastatic HCC, with a median survival of a few months [15], coupled with suboptimal chemotherapy efficacy and inability of patients with poor liver function to tolerate chemotherapy, has resulted in a need for alternative treatment strategies.

## 2. Molecular Pathogenesis of HCC

Two main mechanisms are thought to predominate in the pathogenesis of HCC. The first being cirrhosis after tissue damage resultant from either HBV, HCV infections or toxins such as aflatoxin B and from metabolic causes including obesity and NASH [16, 17]. The second is that of oncogene or tumor suppressor gene mutations [18–23]. Both are associated with abnormalities in cell signaling pathways. Targeting various levels in the signaling cascade may help in both the chemoprevention and the treatment of HCC.

Various signaling pathways have been implicated in HCC, including VEGFR, EGFR, ERK/MAPK, and mTOR, among others [17, 24].

## 3. Vascular Endothelial Growth Factor Receptor (VEGFR) Pathway

HCC is a vascular tumor and is dependent on angiogenesis for growth. Important growth factors include vascular endothelial growth factor (VEGF), platelet-derived growth factor (PDGF), epidermal growth factor (EGF), angiopoietins, and fibroblast growth factors. These induce angiogenic signaling via various pathways, including the activation of the RAF/ERK (extracellular regulated kinase)/MAPK (mitogen-activated protein kinase), mammalian target of rapamycin (mTOR), and WNT-signaling transduction pathways.

Adult hepatocytes are able to upregulate the production of the growth factors listed above following liver damage or injury. This up-regulation is usually transient but poses a problem when it becomes dysregulated in a chronically injured liver, leading to sustained growth signaling [25].

Vascular endothelial growth factor (VEGF) is a primary mediator of angiogenesis in HCC [26, 27]. The upregulation of VEGF and increased expression of VEGFR have been demonstrated in both HCC cell lines and serum of HCC patients [28–32].

The disruption of the VEGFR pathway and targeting growth factors that drive the angiogenic process can thus interrupt effective angiogenesis and have clinical effect in the treatment of HCC. Antiangiogenic drugs such as sorafenib and bevacizumab target different points along the VEGFR pathway.

## 4. Sorafenib

Sorafenib (Nexavar, Bayer HealthCare Pharmaceuticals-Onyx Pharmaceuticals) is an oral multikinase inhibitor. It has potent effects against VEGFR-2, VEGFR-3, and PDGFR and also targets kinases of wild-type B-Raf, mutant V559EB-Raf, and C-Raf [25]. Its main action is thought to be that of competitively inhibiting ATP binding to the catalytic domains of the various kinases [33].

Preclinical experiments in mouse xenograft model of human hepatocellular carcinoma showed that sorafenib had antiproliferative activity and that it reduced tumor angiogenesis and tumor cell signaling as well as increased tumor cell apoptosis [34]. 

A phase II study by Abou-Alfa et al. [35] (see also Table 1) of 137 patients with advanced HCC showed that high pretreatment levels of pERK (phosphorylated extracellular regulated kinase) correlated with a longer time to progression (TTP) following treatment with sorafenib. This suggests that tumors containing higher levels of pERK are more sensitive/responsive to sorafenib and that the Raf/ERK/MEK pathway has an important role in HCC. Significantly, it has also identified pERK as a potential biomarker with predictive significance in HCC.

In this study, 34% of patients achieved stable disease (SD) for at least 16 weeks and 8% achieved partial response (PR) or minor response (MR). The median OS was 9.2 months. Compared to historical controls, the results appear favorable. For example, single-arm studies evaluating combination therapy (cisplatin, interferon, doxorubicin and fluorouracil (PIAF) or doxorubicin plus cisplatin) in HCC patients [36, 37] demonstrated median overall survival (OS) of 8.9 and 7.3 months and SD rates of 28% and 16%, respectively.

Important grade 3/4 adverse events observed included hand-foot skin (HFS) reaction, diarrhea, and fatigue, but they were infrequently dose-limiting. No clinically relevant pharmacokinetic differences between Child-Pugh (CP) Class A and Class B patients were noted, and it is unlikely that any dose adjustment is required when administering sorafenib to these 2 groups of patients.

Of note, 72% of patients were classified as CP Class A and 28% as CP Class B. 17% were HBV positive and 48% were HCV positive.

Two subsequent pivotal studies then led to the approval of sorafenib for the treatment of advanced HCC in the USA and Europe [38, 39].

The Sorafenib Hepatocellular Carcinoma Assessment Randomized Protocol (SHARP) [38] trial by Llovet et al. was concluded early after the second interim analysis showed that advanced HCC patients treated with sorafenib had a significant survival benefit over placebo-treated controls.

This was a multicenter, double-blinded, and placebo-controlled phase 3 trial of 602 patients with advanced HCC with no previous systemic therapy randomized to either 400 mg of sorafenib twice daily or matching placebo. Treatment was continued until the occurrence of both radiologic progression as defined by RECIST [40] and symptomatic progression as defined by the Functional Assessment of Cancer Therapy-Hepatobiliary Symptom Index 8 (FHSI8) questionnaire or the occurrence of either unacceptable adverse events or deaths. 

The results were encouraging, with a median OS of 10.7 months in the sorafenib group versus 7.9 months in the placebo-treated group (hazard ratio in the sorafenib group, 0.69; 95% confidence interval (CI), 0.55 to 0.87; *P* < .001). Although there was no significant difference between the two groups in the median time to symptomatic progression (4.1 months versus 4.9 months, respectively, *P* = .77), the median time to radiologic progression was almost doubled, 5.5 months in the sorafenib group versus 2.8 months in the placebo group (*P* < .001). 7 patients (2%) in the sorafenib group and 2 (1%) in the placebo group had a PR, no patient had a complete response (CR). 

Similar to the phase II trial by Abou-Alfa et al. [35], HFS, diarrhea, and weight loss were the most common side effects in the sorafenib group. Adverse effects reported for patients receiving sorafenib were predominantly grade 1 or 2 in severity and mainly gastrointestinal, dermatologic, or constitutional in nature. In particular, diarrhea, hand-foot skin reactions (HFS), weight loss, alopecia, and anorexia were significantly more common in the sorafenib group compared to the group receiving placebo. Grade 3 adverse effects included diarrhea (8% in sorafenib group versus 2% in placebo group, *P* < .001) and HFS (8% versus <1%, *P* < .001). Except for grade 3 hypophosphatemia (11% versus 2%, *P* < .001), grade 3 or 4 laboratory abnormalities occurred at similar frequencies in both groups. The most common adverse events leading to sorafenib discontinuation were gastrointestinal events (6%), fatigue (5%), and liver dysfunction (5%). The rate of discontinuation of study drug due to adverse events, however, was similar in both groups (38% versus 37%).

This was the first phase III study of a systemic therapy to have shown a survival advantage in patients with advanced HCC. In this group of patients with advanced HCC, the median OS and time to radiologic progression were nearly 3 months longer for patients treated with sorafenib than those given placebo.

This group of patients was carefully selected, with the majority having eastern cooperative oncology group (ECOG) performance status of 0 or 1 and the remainder ECOG 2 status. They were CP Class A. 56% of the patients had HCV.

A second similar study was conducted in Asia with 271 patients with advanced (unresectable or metastatic) HCC [39]. None had prior systemic therapy, and all had CP Class A. This trial had no predefined primary endpoint, and the objective was to assess the efficacy and safety of sorafenib in Asia-Pacific patients with advanced HCC. 

Median OS was 6.5 months (95% CI 5.56–7.56) in patients treated with sorafenib compared to 4.2 months (95% CI 3.75–5.46) in the placebo group, hazard ratio 0.68 (95% CI 0.50–0.93, *P* = .014). Median time to progression (TTP) was 2.8 months in the sorafenib group and 1.4 months in the placebo group. There was no significant difference in the time to symptomatic progression (TTSP) between the two groups.

Like in the previous studies, sorafenib was generally well tolerated with manageable side effects. The most common drug-related adverse events in the sorafenib group were HFS (67 out of 149 patients (45%)), diarrhea (38 of 149 (25.5%)), alopecia (37 of 149 (24.8%)), fatigue (30 of 149 (20.1%)), rash or desquamation (30 of 149 (20.1%)), hypertension (28 of 149 (18.8%)), and anorexia (19 of 149 (12.8%)). These were predominantly grade 1 or 2 adverse events.

 In comparison, overall incidence of HFS was 21% and diarrhea 39% in the SHARP study. In this Asian study, treatment discontinuation due to adverse events was similar in both groups (19.5% versus 13.3%). Dose reductions due to adverse events were required in 30.9% (46 of 149 patients) of patients in the sorafenib group compared to 2.7% (2 of 75) in the placebo group. Most common reasons for dose reductions in the sorafenib group were HFS (11.4%) and diarrhea (7.4%).

Although the absolute survival was greater in the SHARP trial for both study groups, the hazard ratios (HRs) for survival (i.e., reduction in the risk of death associated with sorafenib treatment) was comparable between the two studies (0.68 in the study by Cheng et al. [39] and 0.69 in the SHARP trial [38]). This suggests that there is comparable efficacy for sorafenib in both studies and that there are differences in the patient population in the two studies.

Indeed, at baseline, more patients had extrahepatic spread, greater number of hepatic tumor lesions, poorer ECOG status and higher alpha-fetoprotein (AFP) levels in the study by Cheng et al. than in the SHARP trial. It may well be than the patients enrolled in the former study had more advanced disease than those in the latter, accounting for the difference in the absolute survival for both sorafenib and placebo groups across the two studies. 

However, other significant differences exist between the two studies. As previously stated, etiological factors for HCC in the Asia-Pacific region differ from other regions. For example, 73% of the patients in the study by Cheng et al. had baseline HBV infection and 8.4% had baseline HCV infection, compared with 12% and 30% for HBV and HCV, respectively, in the SHARP trial. There has been some evidence that patients with HBV-associated HCC may have worse prognosis that those with HCV-related HCC [53] and others which suggests sorafenib may be less efficacious in HBV patients [54].

A subset analysis of their patients with HBV infection showed that those treated with sorafenib had longer OS and TTP than those given placebo, and another study showed that the safety profile of sorafenib in HBV patients was similar to the overall study population [55], leading the authors to conclude that sorafenib is just as efficacious in HBV patients.

Subgroup analysis of patients with HCV in the SHARP study showed similar safety profile in the 178 patients with HCV compared to the overall population [56]. Adverse events were mostly predictable and manageable. OS and TTP in this subset of patients were similar to those of the overall study population. These findings support the efficacy and safety results reported in the SHARP trial in patients with HCC and demonstrate a consistent clinical benefit regardless of HCV status. 

Although sorafenib is approved in the USA for the treatment of all unresectable advanced HCC based on the trials above, the results need to be interpreted with caution. In both trials, patients recruited were CP Class A and had relatively good performance status (ECOG 2 or less). These patients were chosen as it was felt liver function impairment associated with CP Class B or C may potentially confound the results of the study. Hence, the effect of sorafenib in patients with poor liver function or decompensated liver disease is still unclear. 

The study by Abou-Alfa et al. [35] suggests no difference in the tolerability of sorafenib in patients with CP Class A or B disease. Updated data from this trial suggests a similar pharmacokinetic and toxicity profile for CP Class A and B patients [57]. 28 out of 137 patients had blood samples analyzed for pharmacokinetics (21 CP A and 7 CP B patients). AUC (0–8) and Cmax were comparable, as were incidence rates for all adverse events and serious adverse events. Elevated bilirubin in this analysis may be related to sorafenib inhibition of UGT1A1 activity. As expected, CP B patients did worse than CP A patients, with more frequent worsening of their liver cirrhosis. It was unclear, though, if this was drug related or due to underlying disease progression. More data is needed to confirm the safety and efficacy of sorafenib in CP B patients.

Pinter et al. [58] also reported a retrospective series evaluating sorafenib in 59 patients, 40% of whom had CP Class B disease and 17% CP Class C disease. The median survival times for these patients with CP Class A, B, and C disease were 8.3, 4.3, and 1.5 months, respectively, leading the authors to conclude that there was no benefit from systemic targeted therapy in patients with very advanced HCC. A phase I and pharmacokinetic study suggested that sorafenib doses should be titrated against the bilirubin levels (an indication of degree of liver dysfunction) and patients with severe liver impairment may not even be able to tolerate attenuated doses [59].

Further studies to evaluate and confirm the benefits and safety of sorafenib in HCC patients with poorer liver function are required. Also the role of sorafenib as an adjuvant therapy after resection or locoregional therapy needs to be studied, as well as the efficacy of combining sorafenib with either chemotherapy or other targeted therapies.

START, a phase II study of the combination of transcatheter arterial chemoembolization (TACE) with sorafenib in Asian patients with unresectable HCC is still ongoing [41]. The second interim analysis of 50 patients evaluable for efficacy showed that 20 (40%) did not require more than 2 TACE procedures. And of these, 18 achieved a CR while 2 had progressive disease. The remainder 30 had PR or SD. Grade 3 adverse events (AEs) occurred in 38 patients (60%), most common of which was hand-foot syndrome. There was 1 grade 4 AE (AST elevation). All AEs improved with sorafenib dose modification, and no patient discontinued due to AE. Preliminary data hence shows that the combination of TACE and sorafenib is safe and tolerable, and further results are awaited.

A phase II trial evaluating the safety and efficacy of doxorubicin plus sorafenib compared to doxorubicin alone in patients with advanced HCC, and CPA disease was conducted by Abou-Alfa and colleagues [42]. In this study, patients were randomly assigned to receive 60 mg/m^2^ of doxorubicin intravenously every 21 days plus 400 mg of either sorafenib or placebo orally twice a day. Ninety-six patients were accrued and following complete accrual, an unplanned early analysis for efficacy was performed and the trial was halted. The median time to progression was 6.4 months in the doxorubicin-sorafenib group and 2.8 months in the doxorubicin-placebo group. PFS was 6.0 months, and 2.7 months and median OS was 13.7 months and 6.5 months in these 2 groups, respectively. Toxicity profiles were similar to those for single agents.

Synergism between sorafenib and doxorubicin is postulated to be the reason behind the improved TTP, OS, and PFS in the group on combined therapy. An ongoing phase III study in advanced HCC patients comparing sorafenib with and without doxorubicin is underway [60]. This combination is as yet not indicated for routine clinical use.

Yau and Chan conducted a phase II trial of sorafenib with capecitabine and oxaliplatin (SECOX) in 51 patients with locally advanced or metastatic hepatocellular carcinoma [61]. In this single-arm, multicentre study, the SECOX regime demonstrates significant clinical activity and good tolerability in this group of patients.

Eighty-four percent of patients were chronic HBV carriers, and 98% had CP A cirrhosis. The best response rate (RR) was 14%, and 61% achieved SD, with median TTP being 7.1 months and OS 10.2 months. Toxicities were mainly grade 1 or 2, with hand-foot syndrome (73%), diarrhea (69%), and neutropenia (63%) being the most commonly encountered. 

Notwithstanding the above studies, sorafenib as single agent remains the only drug so far that has shown overall survival benefit over placebo in a multicentre, double-blind, placebo-controlled randomized phase III trial in patients with advanced HCC [38, 39].

## 5. Bevacizumab

Bevacizumab (Avastin; Genentech, Inc, South San Francisco, CA, USA) is a recombinant humanized monoclonal antibody directed against VEGF [62]. Bevacizumab is also used in the treatment of other malignancies including colon, breast, and kidney cancer. It has been studied both as a single agent, as well as in combination with chemotherapeutic or targeted agents, for example, erlotinib, in the treatment of patients with advanced HCC.

A phase II study of 46 patients using bevacizumab alone in unresectable HCC by Siegel et al. [43] reported a 13% partial response (PR). The 6-month progression-free survival (PFS) was 65%. Overall survival (OS) at 1, 2, and 3 years was 53%, 28%, and 23%, respectively. Grade 3 to 4 adverse events included hypertension (15%) and thrombosis (6%, including 4% with arterial thrombosis). Grade 3 or higher hemorrhage occurred in 11% of patients, including one fatal variceal bleed. 

Bevacizumab was also evaluated in various combinations with chemotherapy including gemcitabine and oxaliplatin [44], capecitabine and oxaliplatin [45] and capecitabine [46].

Zhu et al. showed that combining bevacizumab with gemcitabine and oxaliplatin resulted in a 20% overall response rate in evaluable patients and stable disease in 27%. The median OS was 9.6 months, and median PFS was 5.3 months [44].

A phase II trial performed to evaluate the combination of bevacizumab with capecitabine and oxaliplatin reported a median OS of 10.3 months and a median time to progression (TTP) of 4.5 months. 13.3% (4 out of 30 evaluable patients) had PR and 76.6% (23 patients) had SD [45]. 

Bevacizumab in combination with capecitabine was evaluated in a study by Hsu et al. [46]. Overall response rate was 9% and 52% of patients achieved CR, PR, or SD.

A trial of anti-EGFR therapy (Erlotinib) with bevacizumab is reported below.

## 6. Sunitinib

Sunitinib (Sutent; Pfizer Labs, New York, NY, USA) is another oral tyrosine kinase inhibitor that blocks several receptors, including VEGFR1, 2 and 3, PDGFR-*β*, c-kit, and FLT3 and RET kinase. Most antiangiogenic effects of sunitinib are shown in preclinical studies to be mediated via VEGFR and PDGFR-*β* [63–65]. Sunitinib is being used in the treatment of renal cell carcinoma and gastrointestinal stroma tumor.

In a phase II trial of sunitinib, Zhu et al. [48] showed that that 17 out of 34 patients had SD for at least 12 weeks and 1 had PR. Median progression-free survival (PFS) was 3.9 months and time to progression (TTP) was 4.1 months in this study, in which sunitinib was administered at a dose of 37.5 mg/day. 

In a second phase II study of 37 patients with unresectable HCC, sunitinib (for four weeks out of every six) at 50 mg/day was used. 1 patient achieved PR and 35% had SD. Median PFS was 3.7 months and median OS, 8 months. Significant toxicities, however, were observed, including four deaths. This trial was discontinued early due to low response rate and failure to meet the primary end point [49].

A phase III trial comparing sorafenib with sunitinib was terminated early as a result of a higher incidence of serious adverse events in the sunitinib arm compared to the sorafenib arm and the fact that sunitinib did not meet the criteria to demonstrate that it was either superior or noninferior to sorafenib in the survival of patients with advanced hepatocellular cancer.

## 7. ABT-869

ABT-869 (Linifanib) is an oral tyrosine kinase inhibitor with potent activity against both VEGFR and PDGFR [66]. A phase II open-label, multicenter study of ABT-869 was carried out in 44 patients with unresectable or metastatic HCC [50]. ABT-869 at a dose of 0.25 mg/kg was administered daily to CP A patients and every other day to CP B patients until progressive disease or intolerable toxicity. Of the 34 patients available for analysis, 28 were CP A and 6 CP B. Estimated response rate was 8.7% for 23 CP A patients. Median TTP and PFS for all 34 patients were 112 days, and median OS was 295 days. Most AEs were mild/moderate and reversible with interruption/dose reductions or the discontinuation of ABT-869. ABT-869 appears to benefit HCC patients with an acceptable safety profile. A randomized phase III study in CP A patients with advanced HCC comparing ABT-869 with sorafenib is ongoing [67].

## 8. Brivanib

Brivanib (BMS-582664) is a dual inhibitor of VEGFR and fibroblast growth factor receptor signaling pathways. It has shown tumor inhibitory effects in mouse HCC xenograft models. Raoul et al. [68] conducted a phase II study of brivanib in pts with advanced or metastatic HCC who had no prior systemic therapy (Cohort A) or 1 prior regimen of an angiogenesis inhibitor (Cohort B). 96 patients were enrolled, 55 in Cohort A and 41 (including 38 who failed sorafenib) in Cohort B. In Cohort A, median OS was 10 months and median TTP was 2.8 months. Brivanib appears to have activity as both first-line and second-line post-sorafenib systemic treatment in HCC.

There are ongoing phase III trials assessing brivanib in both first-line setting in comparison with sorafenib as well as in sorafenib-refractory setting in comparison with best supportive care in patients with advanced HCC, and results are awaited [17].

## 9. EGFR and Anti-EGF/EGFR Therapies

EGFR is overexpressed in 40–70% of HCCs [69], and its activation is involved in HCC pathogenesis [70, 71]. EGF is thought to have an important role in tumor angiogenesis, primarily via the activation of the Raf/MEK/ERK and mTOR pathways. The receptor may be targeted via antibodies that block it extracellularly, for example, cetuximab and panitumumab. Intracellular targeting of the EGFR tyrosine kinase with tyrosine kinase inhibitor such as gefitinib and erlotinib are already in use in the treatment of lung and pancreatic tumors [72, 73].

Erlotinib and gefitinib are among some of the tyrosine kinase inhibitors that have shown activity in HCC cell lines and animal models of HCC [74–79].

In a phase II study by Philip et al. [51] of 38 patients with unresectable HCC using single-agent erlotinib, 3 (9%) achieved PR, 12 (32%) were progression-free at 6 months, and the median OS was 13 months. Thomas et al. [52] studied erlotinib alone in 40 patients with CP class A or B advanced HCC. Four months-PFS was 43% and 6 months-PFS was 28%. There was no CR or PR and median OS was 13.3 weeks. 

Combining erlotinib and bevacizumab in a phase II study involving 40 HCC patients, Thomas et al. [47] reported a median PFS of 9 months and an impressive median OS of 15.6 months. 12.5% of the patients had CP Class B disease, and 27.5% had received prior therapy. Side effects included gastrointestinal bleeding (12.5%), fatigue (20%), hypertension (15%). After the initiation of screening for and treating any esophageal varices before being eligible for the study, there were no further episodes of gastrointestinal bleeding. 

An ongoing phase 3 placebo-controlled double-blinded SEARCH (Sorafenib and Erlotinib, a Randomized Trial Protocol for the Treatment of Patients with HCC) trial is being conducted in patients with advanced HCC and CP Class A liver cirrhosis to determine if the OS seen with sorafenib in advanced HCC can be improved by the addition of erlotinib, resulting in combined inhibition of EGF, VEGF, and the RAS/RAF/MEK signaling pathways [80].

Gefitinib (Iressa, Astrazeneca Pharmaceuticals LP, Wilmington DE, USA) has shown activity in preclinical studies in HCC cell lines and animal models, but these results have not been matched in clinical studies. In the study by O'Dwyer et al. [81], single-agent gefitinib showed low activity, with 1 out of 31 patients achieving PR and 7 having SD. Median PFS was 2.8 months, and median OS was 6.5 months. 

Cetuximab (IMC-C225 Erbitux; ImClone LLC, New York, NY and Bristol-Myers Squibb, Princeton, NJ, USA) is a recombinant chimeric monoclonal immunoglobulin 1 antibody targeting the extracellular domain of the EGFR. Similar to gefitinib, however, it has not shown evidence of significant tumor response in HCC. A small study of 30 patients with unresectable or metastatic HCC showed no CRs or PRs, with just 5 patients achieving SD and a median PFS of 1.4 months [82]. Another phase II study by Gruenwald et al. 2007 [83] of single-agent cetuximab in 32 patients showed only limited activity for the drug with a median TTP of 2 months. 

Because of the multilevel receptor cross-stimulation and redundant signaling pathways, it is postulated that just blocking one of these pathways alone may result in others acting as salvage or escape mechanisms for tumor cells. There has been evidence that blocking multiple signaling pathways with a combination of targeted agents may achieve synergistic antitumor effect [84–88]. Most of the anti-EGFR studies being carried out now are thus in combination with cytotoxics or with other targeted agents.

## 10. mTOR Pathway

Several downstream proteins are activated by the EGF and insulin growth factor (IGF) signaling pathways, including phosphoinositide 3-kinase (PI3K), protein kinase B (AKT), and mTOR (mammalian target of rapamycin). expression of both IGF and IGF receptor is upregulated in HCC and human cirrhotic liver [89]. Rapamycin is a natural antibiotic which is a potent inhibitor of mTOR [90]. Three analogues of rapamycin have recently been developed and have been shown to have superior pharmacokinetic and biologic properties.

Sirolimus (Rapamycin) is an mTOR inhibitor with immunosuppressive properties and has been used in the posttransplantation setting. A small pilot study by Rizell and colleagues showed that 6 out of 21 patients had either SD or PR [91].

Temsirolimus is a soluble ester analogue, and everolimus is an orally bioavailable rapamycin derivative. Early clinical trials have shown these agents to have antineoplastic activity, and they are currently being tested in various open clinical trials in the treatment of colorectal, endometrial, and refractory solid tumors [92–94].

There are currently several ongoing phase I and II trials studying temsirolimus and everolimus in patients with advanced HCC, either as a single agent or in combination with another targeted therapy, for example, sorafenib or cytotoxics, for example, pegylated doxorubicin. 

Both rapamycin and everolimus have been shown in xenografts and mouse models to have activity against HCC, either singly or in combination for, example, with sorafenib [95, 96].

Data so far suggests that mTOR inhibitors including the rapamycin analogues are promising agents, and several ongoing trials are exploring this.

## 11. Conclusion

HCC is a complex disease with multiple signaling pathways involved in its pathogenesis. It has proven to be a difficult disease to treat especially in advanced stages. Inhibition of specific growth factor receptors and their various signaling pathways via targeted therapy appears to be a promising approach for the treatment of HCC. More work is required to fully clarify its molecular pathogenesis and to identify other key targets for intervention. 

The use of combination therapy, either with multiple targeted agents or targeted therapy in combination with conventional chemotherapy, may be a more effective way of treating advanced HCC. Combination therapy can target multiple receptors and signaling pathways. Many of these combinations have been shown in preclinical studies to have synergistic effect and may block proposed resistance pathways [97]. Also, fewer overlapping drug toxicities may result when blockade at different pathways via combination therapy is used.

Studies are also underway evaluating vertical as well as horizontal pathway blockade [24]. In vertical blockade, different points along the same pathway are targeted. For example, the use of bevacizumab (VEGF antibody) together with sorafenib (multikinase inhibitor with activity against VEGFR). This may potentially block feedback loops and lead to more complete blockade. In horizontal blockade, however, different signaling pathways are targeted with different drugs, such as the tandem usage of bevacizumab with erlotinib (an EGFR tyrosine kinase inhibitor). Trials combining chemotherapy and other targeted agents with sorafenib are also underway.

Sorafenib was a major breakthrough as an effective targeted treatment in a selected population of patients with advanced HCC. There is an interest in its being used in an adjuvant or neoadjuvant setting in patients undergoing locoregional therapies and even as a chemopreventive in cirrhotic patients.

Other new pathways and molecular targets being investigated include resistance and apoptosis pathways. Also, identifying both predictive and prognostic biomarkers in patients with HCC will be the next step in helping to better tailor HCC treatment.

Much work remains to be done to identify new molecular targets, assess the role of targeted therapy in the adjuvant, neoadjuvant, and metastatic setting, determine the various combinations of treatment, either tandem targeted agents or with conventional cytotoxics, and evaluate the role of sequential versus concurrent therapy.

## Figures and Tables

**Table 1:** 

Agent	Study	Phase	Comparator arm	No. of patients	Response rate	TTP (median months)	OS (median months)	AEs
Sorafenib	Abou-Alfa et al. [35]	II	—	137	34% SD 8% PR/MR	—	9.2	HFS, diarrhea, fatigue

	SHARP trial Llovet et al. [38]	III	Vs placebo	602	2% PR No CR	5.5 versus 2.8	10.7 versus 7.9	HFS (21%), diarrhea (39%)

	Cheng et al. [39]	III	Vs placebo	271	—	2.8 versus 1.4	6.5 versus 4.2	HFS (45%), diarrhea (26%), rash (20%), fatigue (20%)

Sorafenib + TACE	START trial Chung et al. [41]	II	—	50 (evaluable)	36% CR 60% PR/SD	—	—	—

Sorafenib + doxorubicin	Abou-Alfa et al. [42]	II	Vs doxorubicin	96	—	6.4 versus 2.8	13.7 versus 6.5	Same both arms

Bevacizumab	Siegel et al. [43]	II	—	46	13% PR		53% (1 yr) 28% (2 yr) 23% (3 yr)	Hypertens ion (15%), thrombos is (6%)

Bevacizumab + gemcitabine + oxaliplatin	Zhu et al. [44]	II	—	30 (evaluable)	20% RR 27% SD	—	9.6	—

Bevacizumab + capecitabine + oxaliplatin	Sun et al. [45]	II	—	30 (evaluable)	13% PR 77% SD	4.5	10.3	—

Bevacizumab + capecitabine	Hsu et al. [46]	II	—	45	9% RR 52% CR/PR/SD	2.7 (PFS)	5.9	HFS 9% BGIT 9%

Bevacizumab + erlotinib	Thomas et al. [47]	II	—	40	—	9 (PFS)	15.6	BGIT 13%, fatigue 20%, hypertens ion 15%

Sunitinib	Zhu et al. [48]	II	—	34	50% SD	4.1	—	—

	Faivre et al. [49]	II	—	37	2% PR 35% SD	3.7 (PFS)	8	Significant 4 deaths, trial stopped

ABT-69	Toh et al. [50]	II	—	44 (34 evaluable)	8.7% (23 CP A pts)	3.7	9.8	Mostly mild mod

Erlotinib	Philip et al. [51]	II	—	38	9% PR	32% (6 months PFS)	13	—

	Thomas et al. [52]	II	—	40	—	28% (6 months PFS)	3.3	—

RR: overall response rate, MR: minor response, PR: partial response, SD: stable disease, CR: complete response, PFS: progression-free survival, TTP: time to progression, OS: overall survival, AEs: adverse events, HFS: hand-foot syndrome, BGIT: bleeding gastrointestinal tract, CP A: Child Pugh A.
